# Fixed drug eruption and anaphylaxis induced concurrently by erdosteine: a case report

**DOI:** 10.1186/s13223-021-00517-6

**Published:** 2021-02-05

**Authors:** Da Woon Sim, Ji Eun Yu, Young-Il Koh

**Affiliations:** Division of Allergy, Asthma, and Clinical Immunology, Department of Internal Medicine, Chonnam National University Medical School, 42 Jebong-ro, Dong-gu, Gwangju, 61469 Republic of Korea

**Keywords:** Anaphylaxis, Erdosteine, Fixed drug eruption, Hypersensitivity reaction, Simultaneous drug reaction

## Abstract

**Background:**

Erdosteine is used as a mucolytic agent and has a low incidence of adverse drug reactions, most of which are gastrointestinal and mild. Moreover, drug antigens rarely induce multiple simultaneous immunologic reactions. Only one previous case report has demonstrated hypersensitivity reaction induced by erdosteine. Here, we report a case of fixed drug eruption and anaphylaxis, which were concurrently induced by erdosteine. The association between the symptoms and erdosteine was proven by a drug provocation test.

**Case presentation:**

A 35-year-old woman presented with recurrent angioedema and pruritic rash on the hands, which developed within 2 h following the administration of drugs, including erdosteine, for acute upper respiratory infection. Her rash was characterized by well-defined erythematous plaques, which recurred at the same site following the administration of the medications. She also experienced angioedema of the lips. Fixed drug eruption was considered after excluding other possible causes for the presented skin lesions. A drug provocation test confirmed that fixed drug eruption on both hands had occurred after administration of erdosteine, suggesting that erdosteine was the cause of the allergic reaction. However, she also experienced angioedema, isolated wheal, and laryngeal edema; thus, IgE-mediated type I hypersensitivity could also be concurrently occurring with the fixed drug eruption.

**Conclusions:**

We report about a patient who was diagnosed with two different hypersensitivity reactions concurrently induced by erdosteine. We also demonstrate that patients may exhibit multiple simultaneous symptoms that usually arise from overlapping of different hypersensitivity mechanisms. Physicians should be aware of the possibility that some patients who are allergic to certain drugs could exhibit several symptoms caused by different mechanisms of hypersensitivity reactions simultaneously.

## Background

Erdosteine is used as a mucolytic agent because of its ability to enhance mucus clearance and reduce bacterial adhesiveness [1, 2]. Erdosteine is mainly used to treat chronic bronchitis and chronic obstructive pulmonary disease (COPD) and commercialized in 42 countries worldwide [3]. It is also prescribed to treat acute airway infection such as common cold in Korea [4, 5]. Erdosteine is generally safe and well tolerated and is associated with a low incidence of adverse drug reactions, most of which are generally mild reactions of the gastrointestinal system [5, 6].

Drug hypersensitivity reaction is a subset of adverse drug reactions, and drug allergy is a part of drug hypersensitivity reactions for which an immunologically mediated mechanism is demonstrated [7]. Allergic reactions such as drug allergy can be categorized based on their mechanisms of reaction using the Gell and Coomb’s classification [8]. Gell and Coomb’s classification has four groups, namely anaphylaxis type I, type II, type III, and type IV [8]. Type I reaction, which is caused by drug-specific IgE, includes urticaria and anaphylaxis [8]. Type IV reaction is associated with sensitized T cells [8]. Fixed drug eruption (FDE) is considered a localized type IV hypersensitivity reaction, and FDE has several unique clinical manifestations [9]. The most characteristic findings of FDE are recurrence of skin lesions at the same locations and improvement with residual hyperpigmentation [9]. A drug provocation test is the most important method to confirm a culprit drug in FDE [9].

In most cases, single drug antigen induces a single kind of drug allergy with the same mechanism. However, a single drug antigen may also induce multiple immunologic reactions concurrently [8]. Some cases have reported the possibility of combined hypersensitivity reaction types simultaneously induced by one drug [10–12].

Until now, only one such report of erdosteine allergy, which was diagnosed as drug reactions with eosinophilia and systemic symptoms syndrome, was reported in the literature [13]. This means that drug allergy due to erdosteine is rarely observed in the common clinical setting. Thus, physicians may prescribe medications, including erdosteine, to patients who are allergic to them because they usually do not consider erdosteine as the causative agent of drug allergy.

Here, we report the first case of both FDE and anaphylaxis concurrently induced by erdosteine, which was objectively diagnosed using a drug provocation test.

## Case presentation

A 35-year-old woman with recurrent erythematous plaques, lip swelling, hoarseness, and dyspnea presented to our allergy department. Although the physicians changed the prescribed drugs several times, she still experienced adverse reactions many times. She did not have any underlying diseases in need of regular prescription. Furthermore, she had not been diagnosed as having any skin diseases such as urticaria or eczema in the past.

For the past 2 years, the patient experienced suspicious adverse drug reaction after taking medications four times. The first drug reaction occurred after the patient consumed a drug, which was prescribed to another patient for common cold. Unfortunately, she was unaware of the composition of the medication the first time. The patient had taken medications for common cold in the past and did not recall experiencing any adverse drug reactions. The second drug reaction occurred after she took clarithromycin, rebamipide, erdosteine, and codenal syrup (composition: chlorpheniramine maleate + dihydrocodeine bitartrate + dl-methylephedrine hydrochloride + guaifenesin), which were prescribed by a clinician for treating upper respiratory tract infection. After experiencing the second drug reaction, she visited the same clinic and had the physician change the medication. She was then prescribed bepostatine besilate, erdosteine, loxoprofen, pseudoephedrine hydrochloride, and streptokinase-streptodornase. At that time, the physician suspected that clarithromycin was the culprit drug for the adverse drug reaction. However, she still experienced the same reaction after taking the third medication. The fourth drug reaction occurred after taking medication for upper respiratory tract infection that was prescribed by a different physician. She reported that the physician suspected that loxoprofen could be the cause of the drug allergy reaction. She was then administered acetaminophen, bepotastine besilate, erdosteine, and streptokinase-streptodornase. She experienced the same reaction after taking the prescribed medications. She stated that she did not develop any skin lesion spontaneously without taking medication. All skin lesions seemed to be related to the medications.

**Fig. 1:**
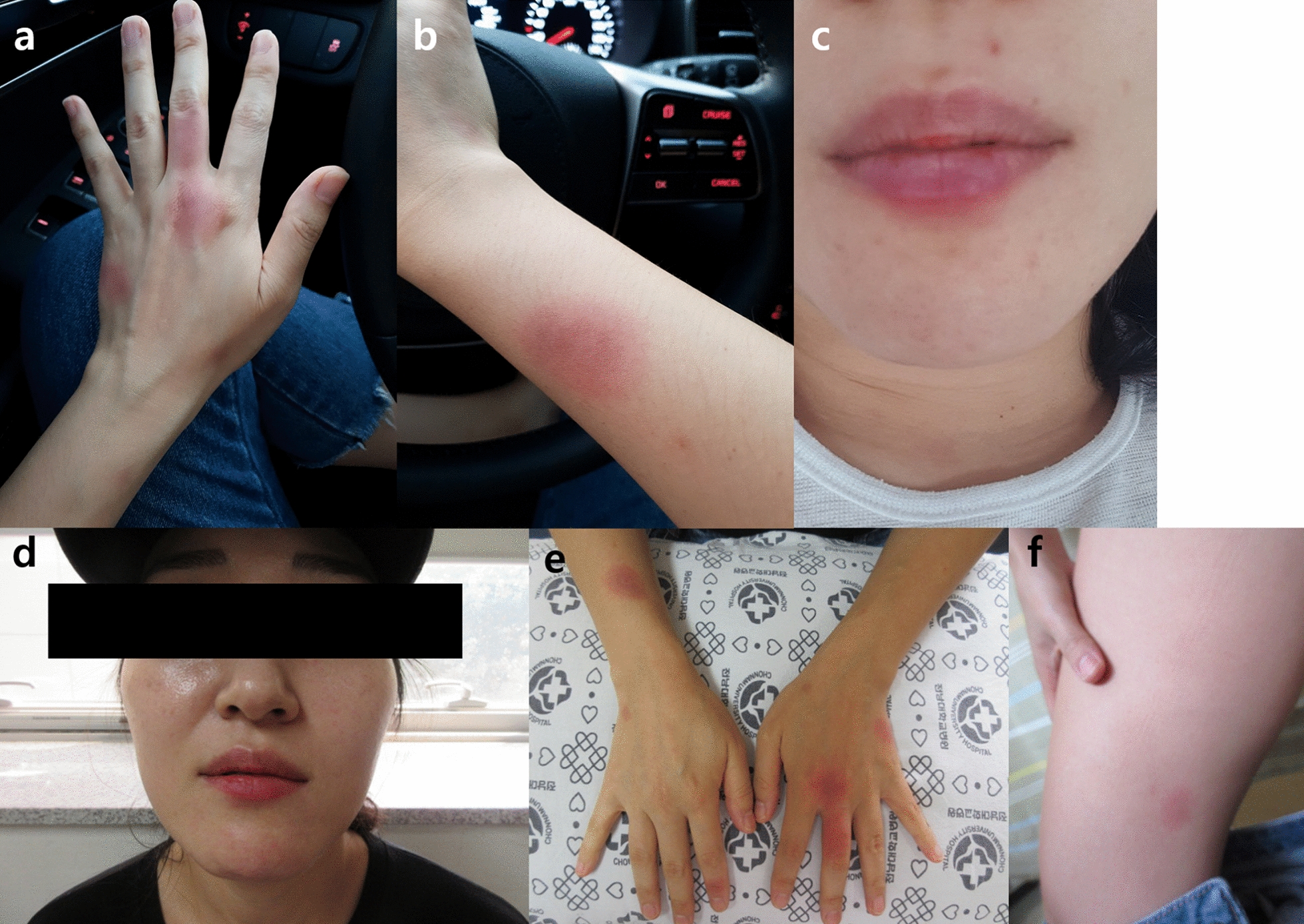
Clinical photographs. **a**–**c** are clinical photos of previous adverse drug reactions and **d**–**f** are clinical photos during the drug provocation test in the allergy clinic. **a**, **b** Erythematous plaques on hand and arm. **c** Lip swelling. **d** Facial and lip swelling. **e** Erythematous plaques on both hands and arm. **f** Isolated wheal on the right thigh

Each time, the patient’s adverse drug reactions symptoms were similar. The reaction started within 2 h after medication. Swelling of the patient’s face and lips occurred, and erythematous plaques appeared on both hands and arms (Fig. 1a–c). She also started experiencing hoarseness and dyspnea. She had pruritic erythematous plaques on both hands, which recurred on the same site but with no pain. The duration of improvement was different for each symptom. Dyspnea and swelling of the face and lip rapidly improved, but the skin lesion resolved only after approximately 7 days after discontinuing the culprit drug. However, she did not have residual hyperpigmentation. During the four reactions, she did not experience hypotension. When the patient visited our allergy clinic, blood test results showed a leukocyte count of 4900/μL, hemoglobin level of 12.3 g/dL, platelet count of 258,000/μL, C-reactive protein level of 0.01 mg/dL, and erythrocyte sedimentation rate of 12 mm/h. Other laboratory test results were normal.

The patient was thought to have a moderate degree anaphylaxis based on her previous history [14]. However, we also suspected FDE because of recurrence at the same site. Although there was no report about anaphylaxis due to erdosteine and the possibility of hypersensitivity reaction due to erdosteine was low, the temporal relationship between the drugs and reactions indicated that the offending drug could be erdosteine. On the Naranjo’s causality assessment scale, the adverse reaction was 8, indicating a “probable” reaction to erdosteine [15], before objective studies were performed. The skin prick test result with erdosteine at a concentration of 30 mg/mL (1/10) was negative on immediate reading. The intradermal test results with erdosteine at 0.3 mg/mL (1/1000), 3 mg/mL (1/100), and 30 mg/mL (1/10) were also negative on immediate reading. To confirm the suspected adverse drug reaction of erdosteine, we conducted a drug provocation test.

In the drug provocation test, 40 min after the administration of 300 mg of erdosteine, she developed pruritus on her lips and finger. After 50 min, she developed lip and facial swelling and several erythematous patch on both hands (Fig. 1d, e). After 60 min, she developed throat tightness and dyspnea. The recorded vital signs were 104/60 mmHg blood pressure, 76 bpm pulse rate, and 97% on room air pulse oximetry. Lung function test results at baseline revealed normal pattern with a forced vital capacity (FVC) of 3.85 L (108%), a forced expiratory volume in one second (FEV1) of 3.43 L (106%) and FEV1/FVC of 82%. Lung function test results after provocation test revealed no significant change with a FVC of 4.25 L (110%), a FEV1 of 3.23 L (99%) and FEV1/FVC of 76%. Skin lesions were composed of several well-demarcated and violaceous plaques, which were surrounded by erythematous concentric circles (Fig. 1e). Moreover, plaques occurred at the same location in the previous exposure, which is classically a FDE manifestation. Simultaneously, she complained of an isolated wheal with pruritus on her right thigh in a position not previously observed (Fig. 1f). Since the skin lesions were typical manifestations of FDE, we did not conduct skin biopsy for excluding other skin disease. We also did not measure the level of serum tryptase, which aids in diagnosing anaphylaxis.

The patient was then administered 0.3 mg epinephrine intramuscular injection and 40 mg methylprednisolone intravenous injection and 4 mg chlorpheniramine maleate intravenous injection. After 1 h of medication, her respiratory symptoms and lip swellings greatly improved. But her skin symptoms on both hands were still present. Seven days after the oral provocation test, the plaques almost disappeared, just like her previous experiences. These results confirmed that erdosteine was responsible for both the anaphylaxis and FDE. She was then prescribed with common cold medication without erdosteine according to our recommendation. The patient no longer has adverse reactions.

## Discussion and conclusions

To the best of our knowledge, this is the first reported case of drug hypersensitivity reaction induced by erdosteine alone. Moreover, this is the first case of FDE and anaphylaxis induced by a single drug that was objectively diagnosed using a drug provocation test. The skin lesions were considered as FDE owing to its clinical course, and respiratory symptoms and lip and facial swelling were manifestations of anaphylaxis.

Only one case of erdosteine-induced hypersensitivity reaction was previously reported [13]. However, the case had limitations. There were two drugs suspected of causing drug hypersensitivity reaction in the patient, and the results of the in vitro tests for the two drugs were almost similar. They only conducted an in vitro test to indirectly prove hypersensitivity reaction due to erdosteine. Drug provocation test is the gold standard for diagnosing drug allergies; however, the patient did not undergo a drug provocation test owing to the severity of the allergic disease. Therefore, the study suggested the possibility of erdosteine as the cause of the hypersensitivity reaction but could not confirm that erdosteine alone was the causative drug.

Our study is the first to prove that the culprit drug was erdosteine alone based on the drug provocation test. In this case, symptoms included recurrent round marginated erythematous plaques on both hands, lip swelling, hoarseness, and dyspnea, which appeared within 2 h following erdosteine administration. In the drug provocation test, skin lesions developed after 50 min of erdosteine administration. FDE commonly appear 30 min to 8 h after the administration of the culprit drug [16]. Skin lesions on both hands were well-demarcated, round to oval, violaceous plaques, which are considered typical skin lesions of FDE [9, 17]. The most important factor in FDE is the same-site recurrence, and this patient developed same-site lesions on both hands during all the adverse drug reaction events [9].

However, there are some aspects of the patient that could not be explained as FDE. Among the symptoms exhibited in this case, lip swelling, hoarseness, and dyspnea are considered clinical features of anaphylaxis [18]. In the drug provocation test, pruritus, lip swelling, throat tightness, and dyspnea were repeatedly observed within 60 min after the administration of erdosteine. Reappearance of symptoms on drug provocation test confirmed that erdosteine induced anaphylaxis [18]. Furthermore, the symptoms rapidly improved after intramuscular injection of epinephrine. The wheal on the right thigh was quite different from the skin lesions on both hands and the location on wheal was not same in previous FDE. Thus, using the drug provocation test, we concluded that two different drug hypersensitivity reactions occurred simultaneously in this patient. Unfortunately, we did not conduct skin biopsy and serum tryptase test; however, tryptase levels and the result of skin biopsy are not always used for the diagnosis of fixed drug eruptions and anaphylaxis. In this case, we could not identify any underlying disease for explaining of these manifestations, and it was it was concluded as FDE and anaphylaxis against erdosteine.

In the literature, there are some reports that reveal that multiple hypersensitivity reactions with different mechanisms can be caused by one drug [10–12, 19, 20]. Only two cases of the occurrence of FDE and anaphylaxis, similar to our case, have been previously described in the literature [19, 20]. Honda et al. reported that autoimmune progesterone dermatitis with urticaria developed into FDE after an intradermal test with progesterone but in the case, patient did not experience anaphylaxis symptoms and FDE concurrently like our case [20]. Sacchelli et al. reported about a patient who developed FDE a month after experiencing anaphylaxis due to the same drug; the patient also did not have anaphylaxis symptoms and FDE concurrently [19]. Moreover, Sacchelli et al. did not provide any objective evidence, such as a provocation test result. In our case, we objectively documented that erdosteine induced both FDE and anaphylaxis. It should be considered that mixed symptoms from multiple hypersensitivity reactions can develop in patients with drug allergy. Further investigation is needed to identify the mechanisms concerned.

This report delineates the case of a patient who developed FDE and anaphylaxis simultaneously caused by erdosteine, which is prescribed for improving upper respiratory tract symptoms. The clinical features indicate type I and type IV hypersensitivity reaction combined; however, the mechanism was unclear because we could not provide evidence to support the hypothesis by experimental results. Future investigation is required to verify the mechanisms involved.

## Data Availability

Yes.

## Abbreviations

COPD: Chronic obstructive pulmonary disease
FDE: Fixed drug eruption
FEV1: Forced expiratory volume in one second
FVC: Forced vital capacity
